# Ligand Binding Modulates the Structural Dynamics and Compactness of the Major Birch Pollen Allergen

**DOI:** 10.1016/j.bpj.2014.10.062

**Published:** 2014-12-16

**Authors:** Sarina Grutsch, Julian E. Fuchs, Regina Freier, Stefan Kofler, Marium Bibi, Claudia Asam, Michael Wallner, Fátima Ferreira, Hans Brandstetter, Klaus R. Liedl, Martin Tollinger

**Affiliations:** 1 Institute of Organic Chemistry, University of Innsbruck, Innsbruck, Austria; 2 Institute of General, Inorganic and Theoretical Chemistry, Center for Molecular Biosciences Innsbruck, University of Innsbruck, Innsbruck, Austria; 3 Department of Molecular Biology, University of Salzburg, Salzburg, Austria

## Abstract

Pathogenesis-related plant proteins of class-10 (PR-10) are essential for storage and transport of small molecules. A prominent member of the PR-10 family, the major birch pollen allergen Bet v 1, is the main cause of spring pollinosis in the temperate climate zone of the northern hemisphere. Bet v 1 binds various ligand molecules to its internal cavity, and immunologic effects of the presence of ligand have been discussed. However, the mechanism of binding has remained elusive. In this study, we show that in solution Bet v 1.0101 is conformationally heterogeneous and cannot be represented by a single structure. NMR relaxation data suggest that structural dynamics are fundamental for ligand access to the protein interior. Complex formation then leads to significant rigidification of the protein along with a compaction of its 3D structure. The data presented herein provide a structural basis for understanding the immunogenic and allergenic potential of ligand binding to Bet v 1 allergens.

## Introduction

**Figure 1:**
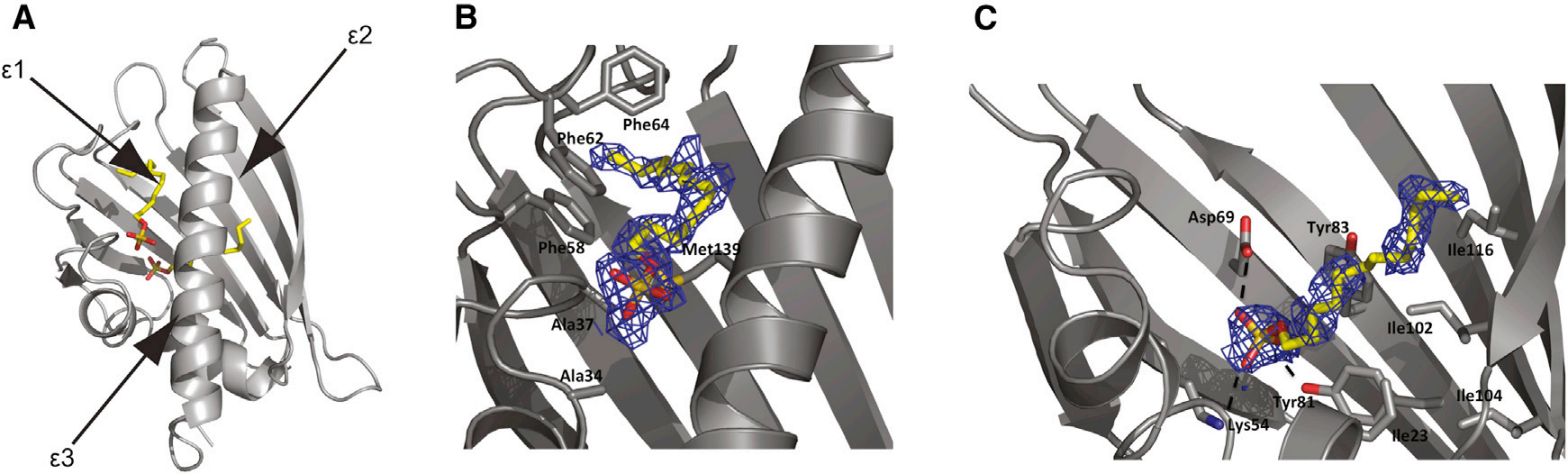
Complex structure of Bet v 1.0101 with two SDS molecules. (*A*) Overview of Bet v 1.0101 in complex with the outer (*front, left*) and inner (*right*) SDS molecules shown in yellow stick representation. Three putative entrances (*ε*1–*ε*3) to the inner hydrophobic cavity are indicated with arrows. (*B*) Zoom-in view of the outer SDS binding site. The ligand molecule in yellow sticks is superimposed on the experimental 2Fo-Fc electron density contoured at 0.8 *σ*. Protein residues that are involved in important interactions with SDS are labeled. (*C*) Zoom-in view of the inner SDS binding site. The ligand molecule in yellow sticks is superimposed on the experimental 2Fo-Fc electron density contoured at 0.8 *σ*. Protein residues that are involved in important interactions with SDS are labeled. To see this figure in color, go online.

The major birch pollen allergen Bet v 1 is a member of the ubiquitous family of pathogenesis-related plant proteins of class-10 (PR-10) (1). PR-10 proteins are known to be involved in defense mechanisms of plants in response to various pathogens, low temperature, oxidative stress, or UV radiation (2, 3, 4). PR-10 proteins consist of ∼160 amino acids that fold into a highly conserved seven-stranded, highly curved, antiparallel *β*-sheet (*β*1–*β*7) along with two consecutive short *α*-helices (*α*1 and *α*2) and a long C-terminal helix (*α*3; Fig. 1) (1).

Together, these structural elements form an extended internal cavity that is capable of binding a variety of physiologically relevant amphipathic ligand molecules (1, 5, 6, 7, 8). It is believed that the biological function of PR-10 proteins involves the storage and transport of phytohormones and other small-molecule ligands.

As a prominent member of the PR-10 protein family, the major birch pollen allergen Bet v 1 has been subjected to a detailed immunological characterization (see www.allergen.org). Bet v 1 is a highly immunogenic protein that represents the main cause of allergic sensitization against birch pollen (9, 10). More than 13 different isoforms of Bet v 1 have been identified, sharing more than 95% sequence identity (11, 12) yet different immunogenic properties, with strikingly different IgE binding capacities in some cases (11). In addition, the 3D structures have been reported for various isoforms of Bet v 1, including the isoform Bet v 1.0101 (Bet v 1a) both with and without ligand(s) bound (8), the single-site mutants M139L (13) and F30V (8) of Bet v 1.0101, the low IgE binding isoform Bet v 1.0106 (Bet v 1j) (8), the naturally occurring hypoallergenic isoform Bet v 1.0107 (Bet v 1l) (5), and Bet v 1.0112 (i.e., the Bet v 1a variant F62L) (14) along with a low IgE binding single-site (E45S) mutant of Bet v 1.0112 (15). Structural data for Bet v 1.0112 are also available for the complex of this protein with a monoclonal murine IgG antibody (16).

The accumulated data for Bet v 1 isoforms and mutants (as well as other members of the PR-10 family) (17) display little variation of the 3D protein structure, with almost identical protein scaffolds (the pairwise root mean-square deviations (RMSDs) between these structures are below ∼1 Å; see Table S1 in the Supporting Material). Notably, even the binding of one or more ligand molecules, such as deoxycholate (DXC), naringenin, and kinetin, to Bet v 1 appears to be accompanied by only minor structural adaptions, predominantly at the protein-ligand interaction surface inside the binding cavity (8). However, ligand binding does have an effect on the immunogenic and allergenic properties of Bet v 1. It was recently shown that binding of DXC to Bet v 1.0101 leads to an increase of the thermodynamic stability of this protein, increases resistance to proteolysis, and affects the binding of human IgE by stabilizing the conformational epitopes on the protein surface (18).

As an additional aspect, from the structural data that are available in the literature to date, it is not evident how exactly ligand molecules enter into the cavity. Three openings to the interior cavity have been identified in crystallographic studies of Bet v 1 proteins (1, 8). These relatively small openings are located between secondary structure elements and might function as gates for solvent water and small ligands (8). It is clear, however, that a certain level of structural dynamics is required for ligand entry, in particular for voluminous molecules such as naringenin, kinetin, and the recently described natural ligand quercetin-3-O-sophoroside (7).

To better elucidate these biophysical features of Bet v 1, a detailed characterization of its structural dynamics is crucial. Here, we characterize the hydrodynamic and dynamic properties of the major Bet v 1 isoform Bet v 1.0101, which constitutes ∼35% of the total protein mass in birch pollen (11), by combining NMR spectroscopic experiments with molecular dynamics (MD) simulations, crystallography, and functional assays. Our hydrodynamic NMR experiments indicate that ligand binding to the interior cavity of Bet v 1.0101 leads to a small but measurable average compaction of the 3D protein structure. The data further show that the conformational space that is dynamically sampled by Bet v 1.0101 when it is bound to ligand molecules is notably different from that sampled by the apo protein. Evidently, compaction of the Bet v 1 structure upon ligand binding is accompanied by a significant rigidification of the protein backbone and the formation of a less dynamic protein. The accumulated data show that these observations are related to the mechanism by which this protein captures and binds ligand molecules. We hypothesize that the rigidification and compaction of Bet v 1 upon ligand binding is related to the observed differences in the proteolytic behavior and IgE binding capacities of the ligand-free and ligand-bound forms of this protein (18).

## Materials and Methods

### NMR sample preparation

Wild-type Bet v 1.0101 protein was prepared as previously described (8, 19). Uniformly ^15^N- (or ^15^N, ^13^C)-labeled Bet v 1.0101 protein was isolated and purified from *Escherichia coli* strain BL21(De3) Star cultures grown in minimal M9 medium enriched with ^15^NH_4_Cl (and ^13^C-glucose). The protein was purified as described elsewhere (19) with some minor changes to the protocol. As a final purification step, size-exclusion chromatography was performed using a 16/60 Superdex75 prep grade column (GE Healthcare Life Sciences, Uppsala, Sweden) with a running buffer of 5 mM sodium phosphate pH 8.0. The concentrations of the protein solutions were determined using NanoPhotometer Pearl (*ε*_0_ (Bet v 1.0101) = 10,430 M^−1^cm^−1^).

### NMR experiments

NMR spectra were recorded at a temperature of 298 K on Agilent DirectDrive 500 and Varian Inova 800 MHz spectrometers. All spectrometers were equipped with room-temperature probe heads. Data were processed using NMRpipe (20) and analyzed using the program CcpNmr (21).Triple-resonance experiments and all relaxation experiments were performed on samples containing 0.4 mM Bet v 1.0101 in 5 mM sodium phosphate pH 8.0 buffer and 8% ^2^H_2_O. All other NMR experiments were performed using 0.2 mM Bet v 1.0101 samples. Experiments with ligands were performed using samples at concentration ratios of Bet v 1.0101/ligand = 1:2 (8-anilino-1-naphthalenesulfonic acid (ANS)), 1:3 (sodium dodecyl sulfate (SDS)), and 1:3 (sodium tetradecyl sulfate (STS)), respectively.

Chemical shift assignments (^1^H, ^15^N, ^13^C’, ^13^C^*α*^, and ^13^C^*β*^) were obtained using the standard 3D triple-resonance experiments HNCACB, HNCO, CBCA(CO)NH, and HNCA. The resonance assignments have been deposited in the Biological Magnetic Resonance Data Bank (http://www.bmrb.wisc.edu) under accession number 25272. To analyze chemical shifts and determine TALOS+ secondary structure probabilities, the program TALOS+ (22) was utilized. Cumulative chemical shift differences, Δ*ω*_cum_, were calculated from ^1^H, ^15^N, ^13^C′, ^13^C^*α*^, and ^13^C^*β*^ chemical shifts as (23)

$$\Delta \omega_{cum}=\sqrt{\frac{1}{N}\sum_{i}{\left(\frac{\Delta \omega_{i}}{\omega_{i,std}}\right)}^{2}},$$

where *N* is the number of nuclei for which chemical shifts are available (≤5), Δ*ω*_i_ is the shift difference of nucleus *i* between ligand-bound and ligand-free states, and *ω*_*i,std*_ is a nucleus-specific normalization factor that takes into account the possible range of chemical shifts (i.e., 1 standard deviation (SD), as observed in the Biological Magnetic Resonance Data Bank).

For pulsed-field-gradient NMR experiments to measure protein translational diffusion, we employed stimulated-echo LED experiments as previously described by Choy et al. (24), with gradient field strengths ranging from 22 to 58 G/cm. For each 2D spectrum, complex data matrices composed of 1024 × 86 points with a maximum acquisition time of 48 ms in the ^15^N dimension were recorded. A total of 32 scans per free induction decay (FID) were obtained with a recycle delay of 1.6 s. The data were apodized using shifted sine bell functions in both dimensions and zero-filled to 512 and 2048 data points. For each spectrum, the intensities (partial peak volumes) of the peaks were obtained by summation in boxes of 5 × 5 data points centered on the peak maximum. The decay of the protein NMR resonances with increasing gradient strength was analyzed as described previously (24) to yield diffusion attenuation rates on a per-residue basis. The average and SD of the attenuation rates of the 20 best-fit residues (as judged by the RMSD) were determined. To estimate the hydrodynamic radii, we performed stimulated-echo LED using dioxane as a standard reference under identical buffer conditions and assumed a hydrodynamic radius of 2.12 Å for dioxane (25). As a control experiment for the negligibility of ligand viscosity, we used 1 mM of the ^15^N-KIX domain of CREB protein in 50 mM potassium phosphate (pH 5.5) and 25 mM NaCl containing 8% ^2^H_2_O and added the same excess of ligand as for the Bet v 1 samples.

Heteronuclear ^1^H, ^15^N NOE, rotating-frame longitudinal relaxation rates R_1*ρ*_, and longitudinal relaxation rates R_1_ were measured for Bet v 1.0101 in the states Bet v 1.0101 (free) and Bet v 1.0101 (SDS-bound) at 298 K and 800 MHz ^1^H Larmor frequency, as previously described (26). The heteronuclear ^1^H, ^15^N NOE was obtained by recording, in an interleaved manner, one spectrum with a delay of 3 s followed by proton saturation for 4 s and another spectrum with a delay of 7 s without proton saturation. Relaxation delays between 10.9 and 870.4 ms were used for R_1_ experiments, and delays between 10.0 and 100.0 ms were used for the R_1*ρ*_ measurements. For each 2D spectrum, complex data matrices composed of 1639 × 90 points with a maximum acquisition time of 31.2 ms in the ^15^N dimension were recorded. Eight scans per FID were obtained with a recycle delay of 1.8 s (R_1_)/1.5 s (R_1*ρ*_). The data were apodized using shifted sine bell functions in both dimensions and zero-filled to 512 and 2048 data points. For each spectrum, the intensities (partial peak volumes) of the peaks were obtained by summation in boxes of 5 × 5 data points centered on the peak maximum. The data were fitted to exponential decays to determine R_1*ρ*_ and R_1_, and R_2_ values were derived from the data as previously described (26). Internal dynamics, along with the rotational correlation time, were analyzed with the program FAST-ModelFree (27). The isotropic rotational correlation time, *τ*_c_, was determined as 8.4 ns and 8.7 ns for SDS-bound Bet v 1.0101 and free Bet v 1.0101, respectively.

^15^N relaxation dispersion experiments were performed at two static magnetic field strengths corresponding to ^1^H Larmor frequencies of 500 MHz and 800 MHz, using Carr-Purcell-Meiboom-Gill (CPMG) pulse sequences described previously (28, 29, 30). Spectra were collected as series of 2D data sets with CPMG field strengths, *ν*_CPMG_ = 1/(2T_CPMG_), where T_CPMG_ is the time between two successive 180° pulses in the CPMG pulse train, between 33 Hz and 933 Hz (with repeat experiments at 67 Hz and 600 Hz), with a relaxation delay set to 30 ms. Spectra were recorded as 1366 × 90 complex points with a maximum acquisition time of 50 ms in the ^15^N dimension. The *t*_1_ (*t*_2_) domain data were apodized using shifted sine bell functions in both dimensions and zero-filled to 512 (2048) data points. Partial peak volumes were obtained by adding the intensities in 5 × 5 grids centered on the peak maximum, and converted to effective relaxation rates via R_2,eff_ = −1/T_relax_ × ln(I/I_0_), where I is the partial peak volume at a given CPMG field strength and I_0_ is the partial peak volume in a reference experiment recorded without T_relax_. The relaxation dispersion data were analyzed by globally fitting the Carver-Richards equation (31) to the experimental data using in-house-written software to extract Δ*ω*_disp_ values.

Titration experiments with SDS, STS, and ANS as ligands were performed by stepwise addition of the ligands (each dissolved in 5 mM sodium phosphate pH 8.0 and 8% ^2^H_2_O) to a 450 *μ*l sample of ^15^N-labeled Bet v 1.0101 to a ratio of 1:2 (ANS) and 1:3 (SDS and STS). Heteronuclear single quantum coherence (HSQC) titration experiments were recorded at 500 MHz. A series of identical ^1^H–^15^N HSQC spectra were performed as complex data matrices composed of 1026 × 80 points, with maximum acquisition times of 128 and 44.4 ms in the ^1^H and ^15^N dimensions, respectively. Sixteen scans per FID were obtained with a recycle delay of 1.4 s, resulting in a total experimental time of ∼1 h for each ^1^H-^15^N HSQC spectrum. For each spectrum, the intensities (partial peak volumes) of the peaks were obtained by summation in boxes of 5 × 5 data points centered at the peak maximum. Chemical shift perturbations (CSPs) caused by the binding of the ligand were plotted against the increasing ligand concentration. Experimental data were fitted to extract the dissociation constant K_d_.

### MD simulations

MD simulations were performed to probe the conformational dynamics of Bet v 1.0101 in the apo and ligand-bound states at atomic resolution. For this purpose, DXC was chosen as ligand because it leaves the interior cavity during the time course of the simulation. We used high-resolution x-ray structures of Bet v 1.0101 in the presence and absence of two molecules of DXC as starting points (Protein Data Bank (PDB) ID: 4A80 and 4A88 (8)). The systems were protonated for physiological pH using the tool protonate3d of MOE (32) and soaked in a truncated octahedral box of TIP3P water molecules (33) with a minimum wall distance of 10 Å. The bound ligand DXC was parameterized using the Generalized Amber Force Field (GAFF) (34). Partial charges were derived from RESP fitting (35) at HF-6/31G^∗^-level using Gaussian03 (36). The applied GAFF parameters for DXC are available from the authors on request. Simulations were performed within the AMBER package using GPU-accelerated PMEMD (37) and the AMBER force field ff99SBildn (38). Thus, we were able to sample a 1 *μ*s unrestrained MD trajectory in the NpT ensemble for both systems after employing a careful equilibration protocol involving several minimization, heating, and cooling steps (39). Simulations were performed at 300 K.

To broaden the phase-space coverage, we performed a replica exchange MD (REMD) simulation (40) of apo Bet v 1.0101 employing the same parameters described above. We ran 32 parallel replica simulations in 2 K temperature spacing spanning a range from 300 to 362 K. The replica were allowed to exchange with a probability of 0.25 while attempting 1000 exchanges over the sampling time of 100 ns. Analyses are presented for the simulation at 300 K only. MD trajectories were analyzed using ptraj and cpptraj of AmberTools (41). After performing standard stability tests via RMSD plots, we analyzed the radius of gyration using all protein atoms as well as B-factors of ^13^C^*α*^ atoms after global alignment to the structure after equilibration.

### Protein crystallization and structure determination

To grow Bet v 1.0101 crystals using the vapor diffusion technique, 5 mg/ml protein (1 *μ*l) was mixed with a precipitation solution consisting of 2.5 M (NH_4_)_2_SO_4_ and 1.5% 2-methyl-2,4-pentanediol. Crystals were obtained by equilibrating the drops against the 100 *μ*l reservoir consisting of the precipitant solution. SDS was added at a 2 mM concentration to the already grown apo crystals for soaking.

Crystals were flash frozen in a nitrogen stream at 100 K, and diffraction data were collected at the ESRF beamline ID14-4. Diffraction data were processed by using the CCP4 software suite (42), solved by Molecular Replacement (43), and refined using REFMAC (44). Due to a severe ice ring at 1.9 Å, x-ray diffraction data were used at 2 Å resolution for refinement. Electron density interpretation and model building were carried out using the program Coot (45). Data collection and refinement statistics are summarized in Table S2. The coordinates of Bet v 1.0101 bound to SDS have been deposited in the Protein Data Bank under entry code 4QIP.

## Results

### NMR diffusion experiments

**Figure 2:**
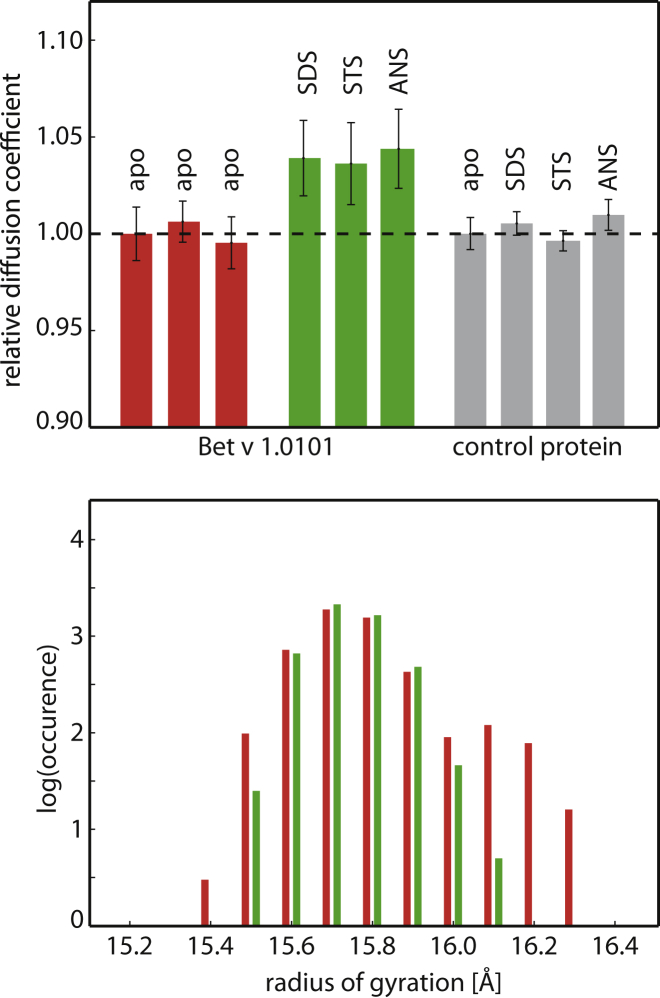
Compactness of Bet v 1.0101. Top: PFG-NMR translational diffusion data. Relative translational diffusion coefficients (D_t_/D_t,apo_) of Bet v 1.0101 (0.2 mM) without ligand (three repeat experiments), and with ligand bound (0.6 mM SDS or STS, 0.4 mM ANS) are shown. Mean values (bars) and SDs (error bars) for 20 backbone amide NH resonances are reported. Control experiments were performed with the CBP KIX domain. Bottom: distribution of the radius of gyration of apo Bet v 1.0101 (*red*) and DXC-bound Bet v 1.0101 (*green*) found in MD simulations. To see this figure in color, go online.

We employed pulsed-field-gradient (PFG) translational diffusion NMR methods to probe the hydrodynamic radius of Bet v 1.0101 without and with ligand bound. As model ligands, we chose SDS, STS, and ANS. SDS and STS both bind to Bet v 1.0101 in a 1:2 stoichiometry (6), with affinities between 1 *μ*M (STS)/7 *μ*M (SDS) and 20 *μ*M (STS)/100 *μ*M (SDS) (Figs. S1 and S2) (6), whereas ANS binds to Bet v 1.0101 in a 1:1 stoichiometry, with ∼19 *μ*M affinity at neutral pH (6). Fig. 2 shows the PFG translational diffusion data for apo Bet v 1.0101 and for Bet v 1.0101 after addition of SDS, STS, or ANS to the protein.

It is evident from this experiment that ligand binding to Bet v 1.0101 has a measurable effect on its translational diffusion behavior. In all three cases (SDS, STS, and ANS), ligand binding accelerates diffusion by roughly 4%. Detergents such as SDS and STS are known to moderately increase the solvent viscosity at mM concentrations. However, under the conditions we used in our experiments (concentration of ligand <1 mM, 298 K) the effect of these ligands on solvent viscosity was negligible (Fig. 2), as shown by control experiments with the CBP KIX domain, which does not bind to SDS, STS, or ANS (see Materials and Methods).

The translational diffusion constant, D_t_, is related to the hydrodynamic radius, r_h_, of a protein by the Stokes-Einstein equation as D_t_ = *k*_B_*T*/6*ρη*r_h_, where *k*_B_ is the Boltzmann constant, *T* is the temperature, and *η* is the solvent viscosity. Thus, for Bet v 1.0101, the observed acceleration of translational diffusion upon ligand binding can clearly be ascribed to a reduction of the hydrodynamic radius of the protein by ≈4%, indicating a compaction of its 3D structure. In absolute numbers, using dioxane as a reference substance for the PFG-NMR diffusion measurements, this means that the hydrodynamic radius of Bet v 1.0101 decreases from ∼20.1 Å to ∼19.3 Å upon ligand binding. The hydrodynamic NMR studies show that ligand binding to the major birch pollen allergen Bet v 1.0101 is accompanied by a measurable degree of compaction of the average protein structure.

### MD simulations

To verify the translational diffusion data, we performed MD simulations of Bet v 1.0101 without ligand bound and in complex with two molecules of DXC (8). The results indicate that ligand binding has an effect on the conformational space that is sampled by the Bet v 1 protein, as is evident from the distributions of the radii of gyration of the conformers present in the simulations (Fig. 2). Specifically, in the apo Bet v 1.0101 REMD simulations (40), a small proportion of protein structures have a higher radius of gyration (up to 16.3 Å) compared with the DXC-bound protein. Therefore, the MD data suggest that more expanded conformers are present in apo Bet v 1.0101, in line with the translational diffusion NMR data. For crystalline Bet v 1, x-ray crystallographic studies so far have indicated that the 3D structure of Bet v 1 proteins in the crystalline state is not affected appreciably by ligand binding (8). Indeed, the radii of gyration of crystal structures that are available in the PDB are all very similar, i.e., between 15.2 and 15.7 Å (Table S1). A solution NMR structural bundle of Bet v 1.0101 in the absence of ligand was reported by Gajhede et al. (14), with a strikingly similar radii of gyration ranging between 15.3 and 15.7 Å.

To calibrate the observed SDS-induced compaction of Bet v 1 with previously reported structural data, we determined the crystal structure of the Bet v 1 with SDS. Consistent with previous reports (6), we find two SDS molecules bound to Bet v 1, which retains a conserved structure as compared with complexes with other ligands or the apo structure of Bet v 1 (8). The inner SDS binding site is harbored by the prominent hydrophobic pocket and the outer SDS binding site is located near entrance *ε*1, delimitated by the C-terminal helix *α*3, as well as the loops connecting helix *α*2 with strand *β*2 and strands *β*3-*β*4 (Fig. 1). The outer SDS is extended and oriented approximately parallel to helix *α*3. The inner SDS molecule is similarly extended and oriented approximately perpendicular to the *β*-sheet, with its sulfate group pointing toward the cavity entrance *ε*3. Both SDS molecules are defined by a diffuse electron density, reflecting the inherent flexibility of the alkane chain and at the same time indicating a preferred binding mode for both SDS molecules. The inner sulfate interacts with the hydroxyl group of Tyr-81 and the carboxylate of Asp-69, whereas the sulfate of the outer SDS is exposed to the solvent. The crystal structure, therefore, allows us to unambiguously assign the inner SDS with a high-affinity binding site (K_d_ = 7 *μ*M) and the outer SDS with a lower-affinity binding site (K_d_ = 100 *μ*M; cf. Figs. S1 and S2).

To probe for structural changes of Bet v 1.0101 in response to ligand binding in solution, we analyzed the NMR chemical shifts of the protein backbone. Chemical shifts are sensitive reporters of the secondary structure of proteins and thus provide probes of structural differences between apo Bet v 1.0101 and complexes (46). A comparison of the chemical shifts of apo Bet v 1.0101 with complex(es) of this protein can therefore provide information about structural changes that accompany ligand binding. Using triple-resonance experiments, we assigned the backbone ^1^H^N^, ^15^N and ^13^C nuclei of Bet v 1.0101 without ligand present and in the presence of saturating amounts of SDS and STS. We did not include ANS-bound Bet v 1.0101 in the chemical shift analysis to avoid the effects on protein chemical shifts that result from the aromatic moiety of ANS.

**Figure 3:**
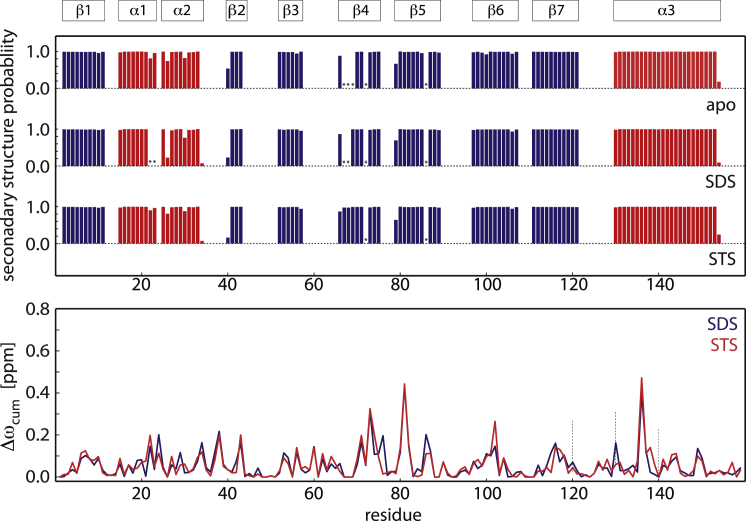
NMR chemical shift data. Top: secondary structure of Bet v 1.0101 as defined by Gajhede et al. (14). Middle: TALOS+ secondary structure probabilities (*blue*, *β*-sheet; *red*, *α*-helix) of Bet v 1.0101 derived from ^1^H^N^, ^15^N, ^13^C’, ^13^C^*α*^, and ^13^C^*β*^ chemical shifts for the apo protein compared with complexes with SDS and STS. For residues that are marked by an asterisk, resonance assignments were not obtained. Bottom: cumulative chemical shift differences, Δ*ω*_cum_, between Bet v 1.0101 in the absence of ligand and Bet v 1.0101 in complex with SDS (*blue*) and STS (*red*), respectively, as a function of residue number. To see this figure in color, go online.

Fig. 3 compares the secondary structures of apo Bet v 1.0101 and Bet v 1.0101 in complex with SDS and STS that were derived from the backbone chemical shifts using the program TALOS+ (22). As expected, the secondary structures closely match the available crystal and NMR structures (8, 14). Notably, there are no appreciable differences between the TALOS+ of the apo protein Bet v 1.0101 and complexes of this protein with SDS or STS, confirming that the secondary structures of apo Bet v 1.0101 and complexes with SDS or STS are very similar. Fig. 3 also shows the cumulative change in backbone ^1^H^N^, ^15^N, ^13^C′, ^13^C^*α*^, and ^13^C^*β*^ chemical shifts, Δ*ω*_cum_, upon binding to SDS and STS, respectively (23). For regions of the protein with little conformational difference between apo and SDS or STS ligand-bound forms, Δ*ω*_cum_ values close to zero are expected, whereas Δ*ω*_cum_ values exceeding ∼1 ppm indicate a structural rearrangement upon ligand binding. For Bet v 1.0101, only minor chemical shift differences between the apo and ligand-bound forms are found, with Δ*ω*_cum_ well below 1 ppm, indicating that ligand binding has only a small effect on the 3D structure of the protein.

Somewhat elevated Δ*ω*_cum_ values (>0.25 ppm) for SDS and STS binding are found for residues in the central *β*-sheet (Glu-73, Tyr-81, and Ile-102) and the C-terminal helix *α*3 (Ser-136). The backbone amide of residue Glu-73 (*β*4) hydrogen bonds to residue 82 of strand *β*5, which is located between two tyrosine residues, Tyr-81 and Tyr-83. The side chains of both Tyr-81 and Tyr-83 are oriented to the inside of the binding cavity, where they interact with bound ligand (Fig. 1), consistent with previously reported crystallographic studies (5, 8). Likewise, the aliphatic side chain of Ile-102 (strand *β*6) points to the interior of the protein cavity, where it interacts with ligands (6, 8), and the backbone carbonyl of Ile-102 is hydrogen bonded to the backbone amide of Tyr-81. Ser-136 is located in a segment of helix *α*3 (between 129 and 132) that is known to adapt its conformation upon ligand binding to Bet v 1.0101 (8). These data are therefore in excellent agreement with the crystallographic mapping of the inner SDS binding site that partially overlaps with the binding site of ANS, DXC, naringenin, kinetin, and other ligands to Bet v 1.0101.

Taken together, the accumulated chemical shift and hydrodynamic NMR data imply that ligand binding to Bet v 1.0101 is accompanied by a slight restructuring and compaction of the protein that does not involve any measurable change of the secondary structure. It should be kept in mind, however, that in structurally heterogeneous proteins, chemical shifts and hydrodynamic NMR data represent averages over the individual structures, so long as the dynamic interconversion of the different conformers that are sampled by the protein is fast on the NMR chemical shift timescale (approximately milliseconds). Therefore, it is critical to characterize the structural dynamics of Bet v 1.0101 in more detail.

### NMR relaxation experiments

**Figure 4:**
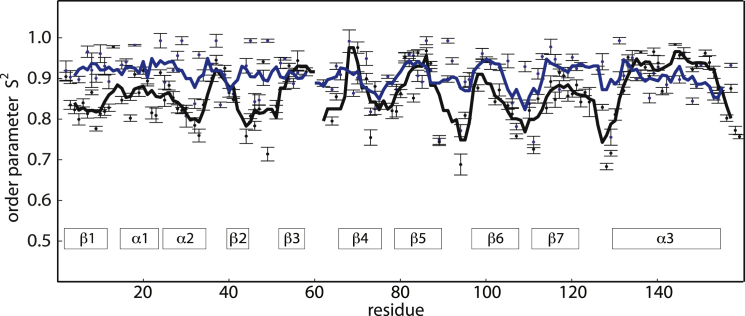
Picosecond–nanosecond dynamics of Bet v 1. Backbone amide ^15^N order parameters S^2^ and error bars of apo (*black*) and SDS-bound Bet v 1.0101 (*blue*) are shown as a function of residue number. Averages of S^2^ (within a sliding window of ±2 residues) are indicated by black and blue lines, respectively. To see this figure in color, go online.

NMR spectroscopy provides various experimental tools for characterizing the structural dynamics of proteins on various timescales (47, 48). The order parameter S^2^, which is accessible from backbone amide relaxation experiments, is sensitive to picosecond–nanosecond dynamics of backbone amide NH groups (47). Fig. 4 compares the experimental backbone NH order parameters of Bet v 1.0101 without and with SDS bound. Extreme S^2^ values of 1 and 0 correspond to fully restricted and unconstrained internal mobility of the backbone amide NH group, respectively. S^2^ values > 0.7 are found for both protein states, which is characteristic for well-ordered and folded proteins.

The order parameter S^2^ versus the residue profile (Fig. 4) provides a strong indication as to which regions of the Bet v 1.0101 protein backbone become more ordered (on the picosecond–nanosecond timescale) upon ligand binding. Interestingly, with the exception of helix *α*3, the S^2^ values for Bet v 1.0101 in complex with SDS are generally higher than those for the apo protein, indicating that the backbone of Bet v 1.0101 becomes more rigid upon complex formation. Rigidification is most prominent for the first strand of the central *β*-sheet (*β*1) and the short helices *α*1 and *α*2, as well as for loops between secondary structure elements (e.g., *β*2/*β*3, *β*5/*β*6, and *β*7/*α*3). Notably, ligand binding does not affect the dynamics of the central part of the *β*-sheet (*β*2−*β*7) and the long helix *α*3 to a similar degree.

From the backbone amide relaxation data, we obtained the rotational correlation time, *τ*_c_, of Bet v 1.0101. We found that ligand binding slightly accelerates rotational diffusion, i.e., the value of *τ*_c_ for SDS-bound Bet v 1.0101 (*τ*_c_ = 8.4 ns) is slightly smaller than that for the ligand-free protein (*τ*_c_ = 8.7 ns). The backbone amide relaxation data are thus consistent with the translational diffusion data, with both indicating a ligand-induced compaction of Bet v 1.

**Figure 5:**
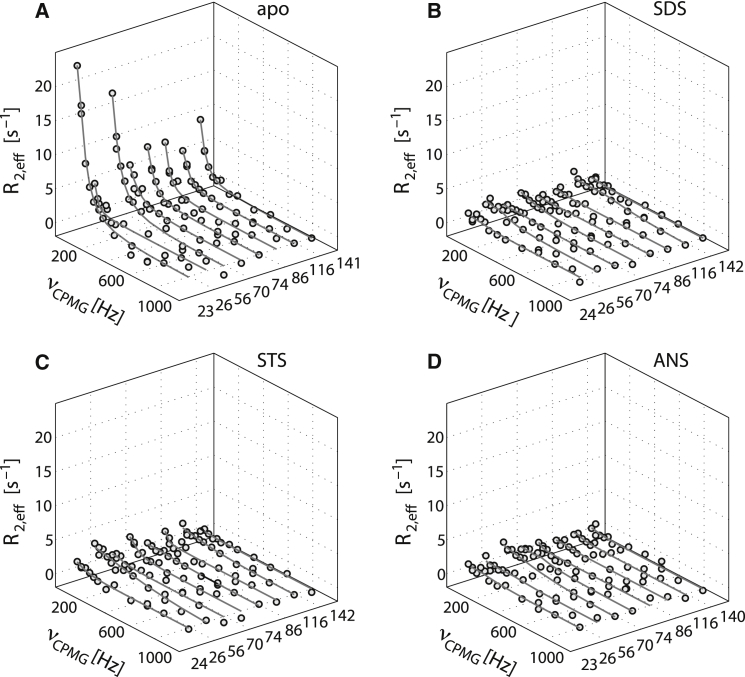
Millisecond timescale dynamics in Bet v 1. (*A–D*) CPMG relaxation dispersion profiles for the ^15^N backbone amides of eight representative residues, along with best-fit lines, of (*A*) apo Bet v 1.0101, (*B*) SDS-bound Bet v 1.0101, (*C*) STS-bound Bet v 1.0101, and (*D*) ANS-bound Bet v 1.0101, recorded at 500 MHz (298 K).

To further probe the structural dynamics of apo Bet v 1.0101 in comparison with Bet v 1.0101 complexes, we performed ^15^N backbone amide relaxation dispersion experiments. These experiments are sensitive to dynamic processes that occur on the microsecond–millisecond timescale, such as transitions between different conformers in structural ensembles (47, 48). Fig. 5 shows representative ^15^N backbone amide relaxation dispersion data for Bet v 1.0101 before and after SDS, STS, and ANS binding.

It is evident from the nonflat relaxation dispersion profiles in the apo protein that Bet v 1.0101 is dynamic on the microsecond–millisecond timescale. Various amino acid residues throughout the protein backbone are involved in this dynamic process, including the helix-loop-helix segment *α*1/*α*2, the central *β*-sheet, and the C-terminal helix *α*3 (Fig. 5). A comparison with the relaxation dispersion data for ligand-bound Bet v 1.0101 reveals a striking difference: irrespective of the nature of the bound ligand (i.e., SDS, STS, or ANS), all backbone amide resonances display flat or almost flat relaxation dispersion profiles. This clearly shows that ligand binding is accompanied by a significant reduction of the conformational heterogeneity and rigidification of the Bet v 1.0101 protein backbone.

**Figure 6:**
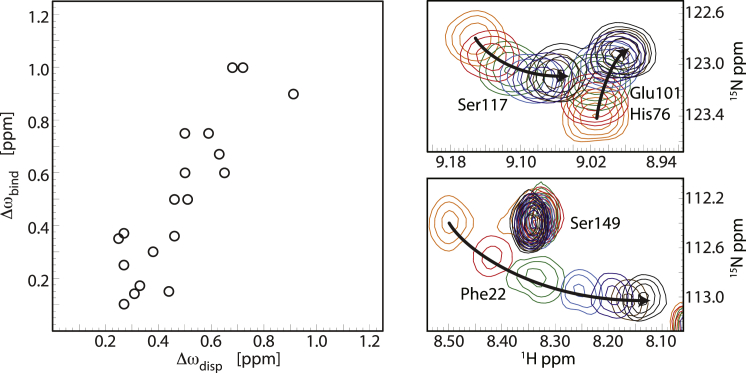
Correlation of relaxation dispersion and titration chemical shifts. Left: plot of the fitted relaxation-dispersion-derived chemical shift differences (Δ*ω*_disp_) versus the measured ^15^N backbone chemical shift differences between apo Bet v 1.0101 and SDS-bound Bet v 1.0101 (Δ*ω*_bind_). Right: ^1^H-^15^N-HSQC titration of Bet v 1.0101 with SDS. Spectra are shown for protein/ligand ratios between 1:0 (*orange*) and 1:3 (*black*). To see this figure in color, go online.

The relaxation dispersion data contain structural information in the form of chemical shift differences between the conformers that are sampled by microsecond–millisecond dynamic processes (47, 48). We performed a quantitative analysis by globally fitting the Carver-Richards equation (30) to the experimental data, which revealed a common exchange process (3.5 ± 0.2 ms) in the intermediate NMR exchange regime and enabled us to reliably determine the chemical shifts. In apo Bet v 1.0101, the variation of the backbone amide ^15^N chemical shifts between the conformers that are present in the structural ensemble are fairly small (<1 ppm), suggesting that they differ only moderately in their backbone conformation. Residues with significant chemical differences between conformers cluster in the helix-loop-helix segment *α*1/*α*2 (Ile-23, Leu-24, Gly-26, Asp-27), in the central *β*-sheet (Ile-56, Arg-70, Val-74, Tyr-83, Ile-86, Ile-103, Lys-115, and Ile-116), and in the C-terminal helix *α*3 (Val-133, Lys-137, and Glu-141). It is noteworthy that all chemical shift variations are well below the values that are expected for unfolding of the protein or a loss of secondary structure (49, 50). Interestingly, however, the chemical shift differences between conformers in the apo protein are comparable to the chemical shift changes we observe for ligand binding using HSQC titrations (Fig. 6). A plot of the backbone amide ^15^N chemical shift differences between apo Bet v 1.0101 and SDS-bound Bet v 1.0101 versus the relaxation-dispersion-derived chemical shift variations between the conformers that are present in the apo protein reveals a roughly linear correlation, with a correlation coefficient of 0.87. This directly demonstrates that the conformational space that is dynamically sampled by Bet v 1.0101 in the absence of ligand already includes conformers that resemble the ligand-bound protein.

## Discussion

Structural studies of PR-10 proteins with and without ligand(s) bound have indicated only a very limited variability of the 3D structure (1, 8, 16). A comparison of the major birch pollen allergen Bet v 1.0101 bound to naringenin, kinetin, ANS, and DXC with the apo protein reveals only minor differences between these structures, irrespective of the exact nature and number of the bound ligand molecule(s) (1, 8). Our dynamic and hydrodynamic data for Bet v 1.0101 point to a distinct difference between the ligand-bound and ligand-free forms of this protein. Bet v 1.0101 displays a measurably higher degree of conformational dynamics on timescales ranging from picoseconds–nanoseconds to microseconds–milliseconds in its apo form than when ligand is bound. Likewise, the hydrodynamic properties of these two forms of Bet v 1.0101 are notably different, indicating that before ligand binding, the 3D structure of this protein is less compact and has a larger average hydrodynamic radius than it does after the ligand is bound to the interior cavity. Taken together, our data suggest that ligand binding to Bet v 1.0101 is accompanied by a defined rigidification of the protein backbone along with a compaction of the 3D structure.

In previous crystallographic studies, it was noted that Bet v 1.0101 has three possible entry points for ligands into the hydrophobic cavity (1, 8). Evidently, however, neither of these entry points is large enough to allow for the passage of voluminous ligand molecules. Therefore, local restructuring of Bet v 1.0101 is a prerequisite for entry of ligand molecules into the binding cavity. It is conceivable that the conformational heterogeneity we observe for apo Bet v 1.0101 is related to the mechanism by which this protein binds ligand molecules. The conformational space that is covered by the apo protein includes conformers that are less compact and have a larger hydrodynamic radius than when ligand is bound. Thus, entrance to the binding sites in the inner hydrophobic pocket could occur predominantly through these larger and more open conformers, where entry of ligand molecules to the internal cavity is alleviated. Ligand binding would then shift the populations of conformers that are present in apo Bet v 1 toward the more compact (closed) ligand-bound state of the protein, which is observed in crystal structures. Concomitantly, the conformational heterogeneity of Bet v 1 would be reduced and a more homogeneous structural ensemble would be observed for the ligand-bound form of the protein.

Such a mechanism of ligand capture and binding is particularly supported by the NMR relaxation dispersion data, which show that conformers that are very similar to the ligand-bound form are already dynamically sampled in the apo form of the protein, i.e., even without ligand being available for binding. Moreover, these data also clearly demonstrate that the level of conformational heterogeneity in Bet v 1 is significantly reduced when ligand is bound, irrespective of the nature of the bound ligand. The rigidification on the microsecond–millisecond timescale is paralleled by a measurable rigidification on the picosecond–nanosecond timescale, as is evident from the NMR order parameters.

It is instructive to analyze the structural details of the dynamic sampling that is present in Bet v 1.0101 before ligand binding. The relaxation dispersion data imply that the millisecond–microsecond structural variations in the apo protein are relatively small, which excludes the disruption of secondary structure elements or unfolding processes. The largest dispersion profiles are found for amino acid residues in the protein interior (the central *β*-sheet and the C-terminal helix *α*3), i.e., close to the ligand-binding site, and in the helix-loop-helix segment *α*1/*α*2. Our data suggest that the millisecond–microsecond dynamic conformational sampling in apo Bet v 1.0101 is most likely related to the conformational selection mechanism by which this protein captures and binds ligand molecules.

This finding is complemented by the observed picosecond–nanosecond timescale dynamics. NMR order parameters show that in apo Bet v 1.0101, dynamics on this timescale is most prominent for parts of the structure (the *β*1-*α*1-*α*2 segment and loops) that connect the central *β*-sheet to the long C-terminal helix *α*3. In Bet v 1 structures, helices *α*1 and *α*2 are arranged in such way that they embrace the C-terminus of helix *α*3 (Fig. 1). Together with strand *β*1, this protein segment constitutes a significant portion of the interaction surface between helix *α*3 and the *β*-sheet. The N-terminus of helix *α*3 is connected to the central *β*-sheet by the long loop between strand *β*7 and helix *α*3, which is also more dynamic in the apo protein than in the ligand-bound form. Thus, it appears that in apo Bet v 1.0101, the interactions between the long C-terminal helix *α*3 and the remainder of the protein are mediated by relatively dynamic structural segments. Ligand binding to the protein interior is accompanied by a rigidification of the interaction surface, which locks the helix into a more rigid arrangement. Such a process could serve to define the position of helix *α*3 with respect to the central *β*-sheet. We hypothesize that the observed rigidification of the Bet v 1.0101 backbone results from stabilizing interactions at the interface between bound ligand and protein.

In a recent study, it was shown that binding of DXC to Bet v 1.0101 stabilizes the protein and increases resistance to proteolysis (18). The thermodynamic stabilities of allergenic proteins are known to play a significant role in the proteolytic processing of these molecules and subsequent presentation of the fragments via the MHC class II complex (51, 52). It is tempting to speculate that for Bet v 1, the less compact conformers that are transiently populated in the apo protein display enhanced susceptibility to proteolytic digestion by exposure of structurally buried amino acid residues. Moreover, the conformational flexibility that we observe for Bet v 1 could contribute to optimal positioning of the epitope residues on the protein surface and facilitate cross-linking of Fc*ε*RI receptors (53). Conformational plasticity is often a prerequisite for efficient recognition of biological targets (54). Taken together, our results provide a possible structural basis for the observed differences in the immunogenic and allergenic properties of Bet v 1 (18).

In summary, we have shown that Bet v 1.0101 without ligand(s) bound displays significantly different dynamic and hydrodynamic properties compared with the ligand-bound protein. Our dynamic NMR data indicate that ligand capture and binding occur by a mechanism for which millisecond–microsecond timescale sampling of different conformers is fundamental.

## Supporting Material

Document S1. Two figures and two tables

Document S2. Article plus Supporting Material
