# Supplementary material for: Ligand Binding Modulates the Structural Dynamics and Compactness of the Major Birch Pollen Allergen
Source: Biophys J. 2014 Dec 16;107(12):2972–81. doi: 10.1016/j.bpj.2014.10.062 (PMC4269767; doi:10.1016/j.bpj.2014.10.062)
Supplement: Document S1. Two figures and two tables [file mmc1.pdf]

SUPPORTING MATERIAL

Ligand Binding Modulates Structural Dynamics and Compactness of The Major Birch Pollen Allergen

Sarina Grutsch, Julian E. Fuchs, Regina Freier, Stefan Kofler,
Marium Bibi, Claudia Asam, Michael Wallner, Fátima Ferreira,
Hans Brandstetter, Klaus R. Liedl and Martin Tollinger

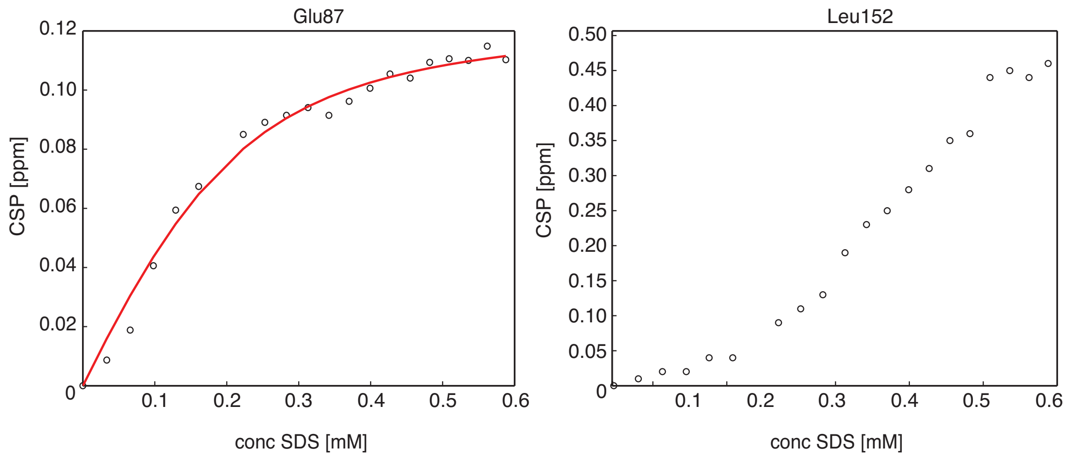

Figure S1: Representative titration curves derived from ^1H-^{15}N HSQC titrations. Left: Weighted chemical shift perturbation (in ppm) observed for the backbone amide resonance of Bet v 1.0101 residue Glu87 upon titration with sodium dodecyl sulfate (SDS)-titration vs. ligand concentration (between 0.03 mM and 0.6 mM, protein concentration: 0.2 mM). The dissociation constant was determined as K_d = 100 µM. Similar titration curves were observed for residues at the primary binding site (see Figure 1C), including Phe22, Arg70, Tyr83 and Ser117. Right: For some residues (e.g., Ala34, Gly140 and Leu152), significant chemical shift perturbations are only observed for protein:ligand ratios exceeding 1:1, indicating the presence of a second ligand binding site (Figure 1B).

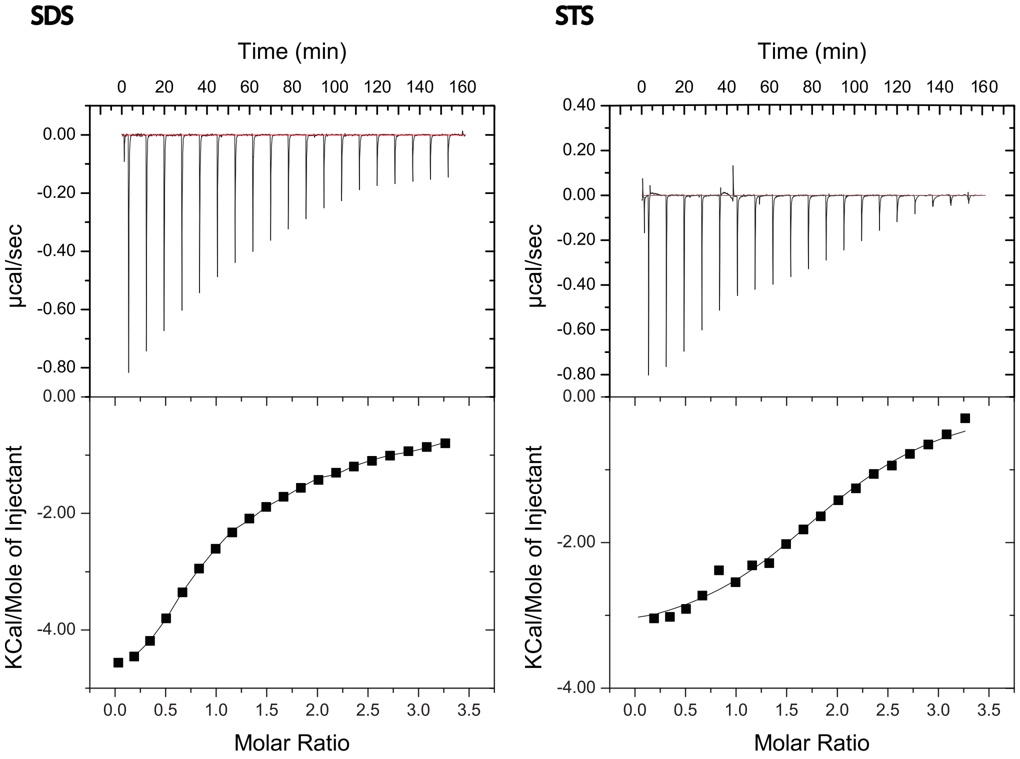

Figure S2. Representative isothermal titration calorimetric data of sodium dodecyl sulfate (SDS) and sodium tetradecyl sulfate (STS) binding to Bet v 1.0101. The heats of binding were measured in a thermogram with 20 injections (2 µl) containing 1.5 mM SDS (right) / 1.5 mM STS (left) into a solution of 94 µM Bet v 1.0101 at 298 K (top: raw thermogram data, bottom: cumulative heat release of reaction is displayed as a function of injection number (binding isotherm)). The stoichiometry and values of K_d are calculated (fitted using two-sites binding model) from these curves to 1 µM (STS) and 7 µM (SDS) for the first and 20 µM (STS) and 100 µM (SDS) for the second ligand.

Table S1. Radii of gyration (R_g) and r.m.s.d. values of Bet v 1 structures

**Ref.**		**PDB ID**	**R_g [Å]**	**r.m.s.d. [Å] (to 1BV1)**
(1)	Bet v 1.0101	4A88	15.47	0.427
(1)	Bet v 1.0101 (complex with DXC)	4A83	15.58	0.497
(1)	Bet v 1.0101 (complex with ANS)	4A80	15.58	0.471
(1)	Bet v 1.0101 (F30V, complex with DXC)	4A84	15.44	0.430
(2)	Bet v 1.0101 (M139L)	1B6F	16.32 [a],[b]	1.558 [b]
(1)	Bet v 1.0106	4A8U	15.45	0.477
(3)	Bet v 1.0107	1FM4	15.66	0.591
(4)	Bet v 1.0112	1BV1	15.67	0
(4)	Bet v 1.0112	1BTV	15.50	1.055
(5)	Bet v 1.0112 (E45S)	1LLT	15.63	0.391
(6)	Bet v 1.0112 (complex with IgG antibody)	1FSK	15.50	0.553

[a] 1B6F with a partially unstructured C-terminus
[b] R_g and r.m.s.d. of first structure in the PDB entry

Table S2. X-ray data collection and model refinement.

**Data collection**	
Wavelength (Å)	0.9393
X-Ray source	Synchrotron
Resolution range (Å)	37.83 - 1.78
Space group	P 1 21 1
Unit cell	32.7 55.6 37.9 90 93.7 90
Total reflections	44009 (5793)
Unique reflections	13028 (1853)
Multiplicity	3.4 (3.1)
Completeness (%)	98.94 (95.60)
Mean I/sigma(I)	8.1 (2.7)
Wilson B-factor	13.8
R-merge	0.082 (0.322)
R-meas	0.097 (0.387)
CC1/2	0.99 (0.88)
CC*	0.99 (0.97)
**Refinement statistics**	
Resolution range (Å)	37.83 - 2.0
R-work	0.244
R-free	0.289
Number of non-hydrogen atoms	1326
Ligands	2
Water	54
Protein residues	159
R.M.S. (bonds)	0.004
R.M.S. (angles)	0.804
Average B-factor	19.6
Ligands	35.0
Solvent	17.8

SUPPORTING REFERENCES

1. Kofler, S., C. Asam, U. Eckhard, M. Wallner, F. Ferreira, and H. Brandstetter. 2012. Crystallographically Mapped Ligand Binding Differs in High and Low IgE Binding Isoforms of Birch Pollen Allergen Bet v 1. J Mol Biol 422:109-123.

2. Schweimer, K., H. Sticht, J. Nerkamp, M. Boehm, M. Breitenbach, S. Vieths, and P. Rosch. 1999. NMR spectroscopy reveals common structural features of the birch pollen allergen Bet v 1 and the cherry allergen Pru a 1. Appl Magn Reson 17:449-464.

3. Markovic-Housley, Z., M. Degano, D. Lamba, E. von Roepenack-Lahaye, S. Clemens, M. Susani, F. Ferreira, O. Scheiner, and H. Breiteneder. 2003. Crystal structure of a hypoallergenic isoform of the major birch pollen allergen Bet v 1 and its likely biological function as a plant steroid carrier. J Mol Biol 325:123-133.

4. Gajhede, M., P. Osmark, F. M. Poulsen, H. Ipsen, J. N. Larsen, R. J. J. vanNeerven, C. Schou, H. Lowenstein, and M. D. Spangfort. 1996. X-ray and NMR structure of Bet v 1, the origin of birch pollen allergy. Nat Struct Biol 3:1040-1045.

5. Spangfort, M. D., O. Mirza, H. Ipsen, R. J. Van Neerven, M. Gajhede, and J. N. Larsen. 2003. Dominating IgE-binding epitope of Bet v 1, the major allergen of birch pollen, characterized by X-ray crystallography and site-directed mutagenesis. Journal of immunology 171:3084-3090.

6. Mirza, O., A. Henriksen, H. Ipsen, J. N. Larsen, M. Wissenbach, M. D. Spangfort, and M. Gajhede. 2000. Dominant epitopes and allergic cross-reactivity: complex formation between a Fab fragment of a monoclonal murine IgG antibody and the major allergen from birch pollen Bet v 1. Journal of immunology 165:331-338.
